# Substrate-Dependent Activation of the *Vibrio cholerae vexAB* RND Efflux System Requires *vexR*

**DOI:** 10.1371/journal.pone.0117890

**Published:** 2015-02-19

**Authors:** Dawn L. Taylor, Vanessa M. Ante, X. Renee Bina, Mondraya F. Howard, James E. Bina

**Affiliations:** University of Pittsburgh School of Medicine, Department of Microbiology and Molecular Genetics, Pittsburgh, Pennsylvania, United States of America

## Abstract

*Vibrio cholerae* encodes six resistance-nodulation-division (RND) efflux systems which function in antimicrobial resistance, virulence factor production, and intestinal colonization. Among the six RND efflux systems, VexAB exhibited broad substrate specificity and played a predominant role in intrinsic antimicrobial resistance. The VexAB system was encoded in an apparent three gene operon that included *vexR*; which encodes an uncharacterized TetR family regulator. In this work we examined the role of *vexR* in *vexRAB* expression. We found that VexR bound to the *vexRAB* promoter and *vexR* deletion resulted in decreased *vexRAB* expression and increased susceptibility to VexAB antimicrobial substrates. Substrate-dependent induction of *vexRAB* was dependent on *vexR* and episomal *vexR* expression provided a growth advantage in the presence of the VexAB substrate deoxycholate. The expression of *vexRAB* increased, in a *vexR*-dependent manner, in response to the loss of RND efflux activity. This suggested that VexAB may function to export intracellular metabolites. Support for this hypothesis was provided by data showing that *vexRAB* was upregulated in several metabolic mutants including tryptophan biosynthetic mutants that were predicted to accumulate indole. In addition, *vexRAB* was found to be upregulated in response to exogenous indole and to contribute to indole resistance. The collective results indicate that *vexR* is required for *vexRAB* expression in response to VexAB substrates and that the VexAB RND efflux system modulates the intracellular levels of metabolites that could otherwise accumulate to toxic levels.

## Introduction

*Vibrio cholerae* is a noninvasive gram negative bacterial pathogen that causes the disease cholera. Cholera is a severe acute diarrheal disease that affects an estimated 3–5 million people per year [1]. Untreated cholera can rapidly lead to dehydration, hypotensive shock, and death. Cholera is contracted by ingesting *V*. *cholerae* contaminated food or water [2]. Following ingestion, *V*. *cholerae* colonizes the small intestine via a process that is dependent upon the induction of genes which are required for intestinal colonization and disease development. These in vivo expressed genes contribute to *V*. *cholerae* pathogenesis in diverse ways and range from traditional virulence factors (e.g. cholera toxin and the toxin co-regulated pilus) to genes that facilitate *V*. *cholerae* survival in the gastrointestinal (GI) tract [3]. Persistence in the intestine is dependent upon *V*. *cholerae’s* ability to overcome antibacterial barriers intrinsic to the GI tract, including the presence of high concentrations of toxic small molecules (such as bile acids and other detergent-like molecules), antimicrobial products generated by resident flora, and products of the innate immune system. In response to these toxic compounds, *V*. *cholerae* activates genes which function to protect the cell by modulating its outer membrane (OM) permeability barrier and by activating efflux transporters [4–6]. For example, in response to bile acids *V*. *cholerae* alters the porin composition of the OM to effectively reduce the rate of bile salt diffusion, and presumably the diffusion of other soluble toxic molecules, across the OM [7–10]. In conjunction with reduced OM permeability, *V*. *cholerae* expresses RND-family transport systems that function to efflux bile salts, and multiple other antimicrobial compounds, from within the cell envelope to the external environment [4–6,11]. Together, the activated RND efflux systems and reduced OM permeability function synergistically to provide *V*. *cholerae* with high-level resistance to lethal antimicrobial compounds present in the host. The importance of these responses in the pathobiology of this organism is highlighted by the fact that *V*. *cholerae* exhibits a greatly diminished ability to colonize the intestinal tract in the absence of these adaptive responses [5,11,12].

The RND efflux systems have been a focal point in bacterial antimicrobial resistance research due to the ability of individual RND systems to provide resistance to a broad range of chemically unrelated substrates that include antibiotics, detergents, dyes, and antimicrobial peptides [13]. The RND efflux systems are found in most gram negative bacteria and function as proton-substrate antiporters [14]. Individual RND efflux systems are composed of three components: an outer membrane pore protein that is homologous to *Escherichia coli* TolC, an integral cytoplasmic membrane pump protein belonging to the RND superfamily, and a periplasmic membrane fusion protein that links the outer membrane pore protein to the RND pump protein [15–18]. Together these three components form a transport apparatus that spans the cell envelope and functions to efflux substrates from within the cell envelope into the external environment. Although the RND transport apparatus is responsible for the efflux of antimicrobials, phylogenetic analysis suggests that the RND efflux systems evolved independent of xenobiotic selection [19,20]. Indeed, there is mounting evidence that the RND efflux systems are involved in diverse functions (reviewed in [21]) such as biofilm formation, iron acquisition, plant-bacteria interactions, lipid transport, bacterial virulence, divalent cation resistance, and the removal of metabolic byproducts from within the cell.

The *V*. *cholerae* genome encodes six RND efflux systems [22]. Inhibition of the RND efflux systems renders *V*. *cholerae* hypersensitive to multiple antimicrobial compounds and attenuates the expression of virulence factors including cholera toxin (CT) and the toxin co-regulated pilus (TCP) [4,23,24]. Among the six RND efflux systems, the VexAB system was shown to be the primary system that contributes to intrinsic antimicrobial resistance in vitro [4,5]. Several studies have suggested that the *V*. *cholerae* VexAB RND efflux system is important to *V*. *cholerae* pathogenesis. This included finding that *vexAB* was induced in vivo in humans and animals [25], that *vexAB* expression was enhanced by the Cpx system [26], and that VexAB was required for high-level virulence factor production [5]. While there is ample evidence to suggest that VexAB is important in pathogenesis, the regulatory mechanisms controlling its expression are unknown.

In many bacteria regulation of the RND efflux systems is mediated by a linked TetR family regulator [27]. The TetR family of regulatory proteins function in diverse phenotypes including antibiotic resistance, metabolism, stress responses, and pathogenicity [28]. TetR proteins contain two functional domains: a conserved N-terminal DNA-binding domain and a variant C-terminal ligand binding domain [28]. The ligand binding domain is capable of binding to effector molecules that modulate the interaction of the DNA binding domain with its target sequences. In the case of RND efflux systems, the activity of the RND efflux system’s TetR regulators is often modulated by the binding of efflux substrates of the linked RND system. In many cases, TetR proteins appear to be capable of binding a diverse set of ligands that correspond to the multiple substrates of linked RND efflux systems [28]. The vast majority of reported TetR family regulators function as repressors that bind the promoter region and repress transcription in the absence of bound ligands [29,30]. In addition to regulating their specific target genes, many TetR regulators also regulate their own expression [27,28].

In this work we tested the hypothesis that the TetR family protein VexR regulated the expression of the *vexAB* RND efflux system [22,31,32]. VexR, which has not been characterized, was encoded by the first gene in a three gene operon that included *vexA* and *vexB*; a genetic organization that was distinct from most RND efflux systems [28,29]. Our results confirmed that VexR functioned in the regulation of the *vexRAB* operon, but in a manner that was opposite to most RND efflux system associated TetR regulators. Our results indicated that VexR was required for activation of the *vexRAB* operon, whereas most RND-linked TetR regulators function as repressors. We further found that the *vexRAB* promoter was upregulated, in a *vexR*-dependent manner, in response to the loss of RND efflux activity. This suggested that endogenous metabolites may serve as activators of the *vexRAB* efflux operon and could be VexAB substrates. Consistent with this idea, *vexRAB* was upregulated in several metabolic mutants, including tryptophan biosynthetic mutants. Furthermore indole, an intermediate in tryptophan biosynthesis, induced *vexRAB* expression. Taken together our results suggested that VexR was required for activation of the *vexRAB* operon. We further posit that a native role of the VexAB RND efflux system is to remove excess cellular metabolites from within the cell that could otherwise accumulate to toxic levels.

## Materials and Methods

### Bacterial strains and culture conditions

The bacterial strains used in this study are listed in Table 1. *Escherichia coli* EC100D*pir*+, SM10λ*pir*, and ER2566 were used for cloning, plasmid mobilization, and protein purification, respectively. *Vibrio cholerae* strains used in this study were derivatives of O1 El Tor strains N16961 and C6706 [22,33,34]. *V*. *cholerae* strain JB58 (N16961-Δ*lacZ* Sm^R^) or JB804 (C6706-*lacZ*+ Sm^R^) were used as wild-type (WT) control strains as indicated. The C6706 transposon mutants [33] were graciously supplied by Dr. John Mekalanos (Harvard Medical School). Bacterial strains were grown at 37°C in Luria-Bertani (LB) broth or on LB agar. AKI broth was used for virulence inducing conditions as described previously [5]. Bacterial stocks were maintained at -80°C in LB broth containing 25% glycerol. Growth media was supplemented with carbenicillin (Cb) and streptomycin (Sm) at 100 μg/mL, kanamycin (Km) at 50 μg/mL, or chloramphenicol (Cm) at 1μg/mL (for *V*. *cholerae*) and 20 μg/mL (for *E*. *coli*) as required. Bacterial growth media was purchased from Difco (Lawrence, KS) and chemicals were purchased from Sigma-Aldrich (St Louis, MO).

**Table 1:** Strains, plasmids and oligonucleotides.

**Strain:**	**Genotype:**	**Source:**
Vibrio cholerae		
JB804	01 El Tor strain C6706, Sm^r^	[34]
JB3	01 El Tor strain N16961, Sm^r^	[4]
JB58	01 El Tor strain N16961 Δ*lacZ*, Sm^r^	[4]
JB114	JB58 Δ*vexM*	[5]
JB116	JB58 Δ*vexH*	[5]
JB432	JB58 Δ*vexF*	[5]
JB464	JB58 Δ*vexD* Δ*vexF* Δ*vexH* Δ*vexK* Δ*vexM*	[5]
JB485	JB58 Δ*vexB* Δ*vexD* Δ*vexF* Δ*vexH* Δ*vexK* Δ*vexM*	[5]
JB495	JB58 Δ*vexB*	[4]
JB528	JB58 Δ*vexK*	[5]
JB692	JB58 Δ*vexD*	[4]
JB694	JB58 Δ*vexB* Δ*vexD*	[4]
JB718	JB58 Δ*vexR* Δ*vexD*	This study
XBV218	JB58 Δ*vexR*	This study
XBV220	JB58 Δ*vexR* Δ*vexB* Δ*vexD* Δ*vexF* Δ*vexH* Δ*vexK* Δ*vexM*	This study
MKW589	Δ*cpxR lacZ::cpxP-lacZ*	[26]
DT1458	JB58 *lacZ::cpxP-lacZ*	[26]
DT1572	JB58Δ*vexB lacZ::cpxP-lacZ*	[26]
DT1460	JB485 *lacZ::cpxP-lacZ*	[26]
DT1693	JB495 *lacZ::cpxP-lacZ*	This study
VA367	JB58 Δ*vexR* Δ*cpxR lacZ::cpxP-lacZ*	This study
EC8508	C6706 Tn::VC0027(*ilvA*)	[33]
EC20412	C6706 Tn::VC0051(*purK*)	[33]
EC1769	C6706 Tn::VC0052(*purE*)	[33]
EC23411	C6706 Tn::VC0164(*vexB*)	[33]
EC24273	C6706 Tn::VC0374(*pgi*)	[33]
EC14462	C6706 Tn::VC0384(*cysJ*)	[33]
EC5082	C6706 Tn::VC0385(*cysI*)	[33]
EC9587	C6706 Tn::VC0386(*cysH*)	[33]
EC19978	C6706 Tn::VC0537(*cysM*)	[33]
EC11960	C6706 Tn::VC0767(*guaB*)	[33]
EC4709	C6706 Tn::VC0774	[33]
EC11507	C6706 Tn::VC0819(*aldA-1*)	[33]
EC8862	C6706 Tn::VC0923	[33]
EC11848	C6706 Tn::VC0968(*cysK*)	[33]
EC10232	C6706 Tn::VC1061	[33]
EC5818	C6706 Tn::VC1169(*trpA*)	[33]
EC24412	C6706 Tn::VC1170(*trpB*)	[33]
EC12331	C6706 Tn::VC1171(*trpC/F*)	[33]
EC11883	C6706 Tn::VC1172(*trpD*)	[33]
EC11883	C6706 Tn::VC1173(*trpG*)	[33]
EC11131	C6706 Tn::VC1174(*trpE*)	[33]
EC1872	C6706 Tn::VC1579	[33]
EC389	C6706 Tn::VC1732(*aroA*)	[33]
EC14803	C6706 Tn::VC1819(*aldA-2*)	[33]
EC12803	C6706 Tn::VC2013(*ptsG*)	[33]
EC7541	C6706 Tn::VC2092(*gltA*)	[33]
EC18511	C6706 Tn::VC2209(*vibF*)	[33]
EC10553	C6706 Tn::VC2348(*deoB*)	[33]
EC24541	C6706 Tn::VC2362(*thrC*)	[33]
EC19558	C6706 Tn::VC2363(*thrB*)	[33]
EC13310	C6706 Tn::VC2364(*thrA*)	[33]
EC1335	C6706 Tn::VC2558(*cysC*)	[33]
EC13560	C6706 Tn::VC2559(*cysN*)	[33]
EC21282	C6706 Tn::VC2560(*cysD*)	[33]
EC9914	C6706 Tn::VC2649(*cysE*)	[33]
EC20144	C6706 Tn::VCA0013(*malP*)	[33]
EC2460	C6706 Tn::VCA0014(*malQ*)	[33]
EC4499	C6706 Tn::VCA0765(*ybjU*)	[33]
EC9834	C6706 Tn::VCA0886(*kbl*)	[33]
EC3123	C6706 Tn::VCA0896(*zwf*)	[33]
EC14445	C6706 Tn::VCA0987(*ppsA*)	[33]
EC21873	C6706 Tn::VCA1046(*mtlD*)	[33]
Escherichia coli		
EC100D*pir*+	F^-^ mcrA Δ(mrr-hsdRMS-mcrBC) Φ80dlacZΔM15 ΔlacX74 recA1 endA1 araD139 Δ(ara, leu)7697 galU galK λ^-^ rpsL (Sm^R^) nupG pir+	Epicentre
SM10λ*pir*	*thi-1 thr leu tonA lacY supE recA::*RP4-2-4-Tc::Mu Km^R^ (λ *pir*R6K)	[62]
ER2566	F- glnV44(AS) galK2(Oc) rpsL704(strR) xylA5 mtl-1 argE3(Oc) thiE1 tfr-3 λ DE3 = λ sBamHIo ∆EcoRI-B int::(lacI::PlacUV5::T7 gene1) i21 Δnin5	New England BioLabs
Plasmids:	Description:	
pBAD18	Expression plasmid, Cb^R^, pBR322 origin of replication	[63]
pCM10	Vector for construction of *lux*CDABE transcriptional fusions, Km^R^, ori101	[64]
pDT1076	pCM10 containing the *vexR* promoter region from N16961	This study
pDT1146	pMMB66EH::*vexR*	This study
pDT1777	pDT1076 with Cm-mark cassette inserted into the vector, Cm^R^	This study
pJB703	pBAD18::*vexR*	This study
pMAL-c2	Expression plasmid for fusion of proteins to MBP and cytoplasmic expression, Cb^R^, pBR322 origin of replication	New England BioLabs
pMMB66EH	Expression plasmid, Cb^R^, oriV/T	[7]
pSC137	Vector for transposon mutagenesis of bacteria, Cm^R^, oriR6K	
pMAL-c2::*vexR*	*vexR* cloned into pMAL-c2	This study
pTL61T	Vector for construction of *lac*Z transcriptional fusions, Cb^R^, oriRK2	[12]
pWM91	Suicide plasmid vector used for allelic exchange, Cb^R^, oriR6K/fl	[65]
pWM91::Δ*vexR*	pWM91::Δ*vexR*	This study
pXB233	pTL61T containing the *vexR* promoter region from N16961	This study
pXB228	pTL61T containing the *vexEF* promoter region	[26]
pXB229	pTL61T containing the *vexGH* promoter region	[26]
pXB230	pTL61T containing the *vexIJK* promoter region	[26]
pXB231	pTL61T containing the *vexCD* promoter region	[26]
pXB232	pTL61T containing the *vexLM* promoter region	[26]
pΔR	*cpxR*::Km allelic exchange vector	[37]
pJL1P’Z	Allelic exchange vector for placing *cpxP-lacZ* into the *V. cholerae* genome	[37]
Oligonucleotides:	DNA sequence (5’–3’)::	
166b-F-XhoI	AACTCGAGGCAGAGAAATGTGATGT	
166b-R-XbaI	AATCTAGAGCCAAACAGCAGGATCG	
166c-F-XhoI	TTCTCGAGGGGTCCGGAGACGTACT	
166c-R-XbaI	CGTCTAGAGGAGCTGTTTATCGCCG	
Biotin	GCGGGAGTCGGCAGCG	
MCS4.VexA.R	CCGGATCCCATTCTGGTGCGAACTCCAAATTAGTGTTG	
VC0166-SacI-F	CTGAGCTCAAGGGTTCATATGCA	
VC0166-XbaI-R	TTTCTAGATTAGTGTTGAGTAATTGCA	
VC0166-F1	CAGGATCCACTTTAGCACCGTTACTCAG	
VC0166-F2	TCATTGCATCCTGTTTATCGCCGTACACTATTTC	
VC0166-R1	ACCTCGAGTATTGGCCAGTATGACCTTG	
VC0166-R2	CGATAAACAGGATGCAATGATTCAAGCCAGTTGG	
VC0166-F-pMAL-SmaI	GGCCCGGGTTGCAGAGAAATGTGATGTCTGAAATAGTG	
VC0166-R-pMal-EcoR1	GGAATTCTTAGTGTTGAGTAATTGCATCC	
*vexR*-F1	GCGGGAGTCGGCAGCGATAATAATCCGCTCACCGAG	
*vexR*-R1	GCGGGAGTCGGCAGCGCCCCTGTTTTGCAATACACTTG	
*vexR*-F2	GCGGGAGTCGGCAGCGTGCAAAACAGGGGGTATTAG	
*vexR*-R2	GCGGGAGTCGGCAGCGGCCGTACACTATTTCAGACA	
XWL-BRL-F	CGCAGGGTTTTCCCAGTCACGAC	

Growth curves were generated as follows. The indicated strains were grown overnight in LB broth and normalized to an OD_600_ of 1.0. The cultures were then diluted 1:20,000 into LB broth with or without arabinose and/or deoxycholate as indicated. Aliquots of 100μL of each strain were then distributed in triplicate into the wells of a flat bottom 96-well microtiter plate. The 96-well plate was then incubated in a BioTek ELx808 plate reader at 37°C with intermittent shaking and growth was monitored by measuring the absorbance at 600 nm every 20 min for 14h. The data was then averaged for the triplicate samples at each time point.

### Plasmid and mutant construction

Plasmids and oligonucleotides used in this study are listed in Table 1. Enzymes for cloning experiments were purchased from New England Biolabs (Beverly, MA). pXB233 (P_*vexRAB-lacZ*_) was constructed as follows. The 166c-F-XhoI and 166c-R-XbaI PCR primers were used to amplify the *vexRAB* promoter from N16961 genomic DNA. The resulting PCR amplicon was then restricted with XhoI and XbaI endonucleases before being ligated into the XhoI/XbaI site of pTL61T to generate pXB233 (P_*vexRAB-lacZ*_). pDT1076 (P_*vexRAB-lux*_) was generated by cloning the *vexRAB* promoter from pXB233 into pCM10 as follows. The *vexRAB* promoter was PCR amplified from pXB233 using the XWL-BRL-F and166c-F-XhoI PCR primers. The resulting PCR amplicon was then made blunt-ended before being restricted with BamHI. The resulting fragment was then cloned into pCM10 linearized with EcoRI, and then blunt-ended, before being restricted with BamHI. A Cm-marked version of pDT1076 was generated by transposing the Cm-marked mariner transposon from pSC137 into pDT1076 to yield pDT1777. All plasmids were verified by DNA sequencing.

The *vexR* expression plasmids were constructed as follows. pJB703 was generated by amplifying *vexR* from N16961 using the VC0166F-SacI and VC0166-XbaI-R PCR primers. The resulting amplicon was restricted with SacI and XbaI endonucleases before being ligated with similarly digested pBAD18. pDT1146 (pMMB66EH::*vexR*) was generated by amplifying the *vexR* gene from N16961 using the VC0166F-SacI and MCS4.VexA.R primers. The resulting amplicon was blunt-ended, digested with BamHI, and then ligated with similarly treated pMMB66EH to generate pDT1146. The sequence of *vexR* in both plasmids was confirmed by DNA sequencing. pMAL-c2-*vexR* was constructed by amplifying the *vexR* gene from N16961 using the VC0166-F-pMAL-SmaI and VC0166-R-pMal-EcoRI PCR primers. The resulting PCR amplicon was then digested with EcoRI and SmaI endonucleases and cloned into the same sites of pMAL-c2 (New England Biolabs).

The allelic exchange vector pWM91::Δ*vexR* was generated by crossover PCR as previously described [4,5,35,36]. Briefly, *vexR*-specific VC0166-F1/-R2 and VC0166-F2/-R1 PCR primer pairs (Table 1) were used in separate PCR reactions with N16961 genomic DNA as a template. The resulting ~1.1 Kb PCR products were gel purified, pooled, and then used as the template for a second PCR reaction using the flanking VC0166-F1/-R1 PCR primers to generate the ~2.2 Kb *vexR* deletion construct. The resulting ~2.2 Kb PCR amplicon was then purified, restricted with XhoI and BamHI endonucleases before being ligated with similarly digested pWM91 to generate pWM91::Δ*vexR*. The resulting plasmid was then used to delete *vexR*. Briefly, pWM91::Δ*vexR* was conjugated into the *V*. *cholerae* and cointegrants were selected for Cb/Sm resistance. Several Cb/Sm resistant colonies were then streaked for single colonies onto LB agar (without NaCl) containing 5% sucrose to select for loss of the integrated plasmid. Several sucrose-resistant colonies were screened for Cb sensitivity to verify plasmid loss before the *vexR* deletion was confirmed by PCR using flanking primers.

Deletion of *cpxR* was accomplished by allelic exchange using pΔR as previously described [26,37]. The *cpxP-lacZ* chromosomal reporter was introduced into the *lacZ* locus using pJL1P’Z as previously described [26,37].

### Reporter assays

The β-galactosidase assays were performed as follows. Test strains were grown in LB broth or under AKI conditions and culture aliquots were taken in triplicate at the indicated time points post-inoculation to quantify β-galactosidase activity as previously described [7]. All experiments were performed at least three times and the results averaged. Statistical significance was determined using ANOVA with indicated post-test.

The bioluminescence reporter assays were performed as follows. *E*. *coli* EC100D*pir+* containing pDT1041 and pDT1124 (or pBAD18) were grown overnight in LB broth before being diluted 1:200 into fresh LB broth with or without 0.2% arabinose. Aliquots (100μL) of the diluted cultures were then distributed in triplicate into the wells of white 96-well microtiter plates with clear bottoms (Corning) and incubated with shaking at 37°C for the duration of the assay. Luminescence and the OD_600_ at indicated time points were determined using a BioTek Synergy HT plate reader. The reported results are the average relative light units (RLU) for each test sample divided by the optical density.

Quantification of *vexRAB* expression in the C6706 transposon mutants was accomplished using pDT1777 (*vexRAB-lux*) as follows. Strains grown in LB broth were diluted 1:200 from the overnight culture into fresh LB broth with Cm and grown under the same conditions as listed above. Strains grown under AKI conditions were diluted 1:10,000 into AKI broth with Cm. Aliquots (370μL) of the diluted cultures were then distributed in triplicate as described above. Plates were incubated statically at 37°C for 4h, at which point 215μL of culture was removed from each well for a final volume of 155μL/well. Plates were then grown with shaking at 37°C for the remainder of the assay. Luminescence production was determined as described above. The results are the average RLUs for each test sample divided by the optical density. Two-way ANOVA with Dunnet’s post-hoc test was used to determine significance relative to WT.

### Antimicrobial susceptibility assays

Antimicrobial susceptibility tests were performed using gradient agar plates as previously described [5]. Each 9 cm x 9 cm gradient plate was inoculated with six *V*. *cholerae* strains, including WT which served as an internal control. The plates were then incubated overnight at 37°C before growth was measured. The minimum inhibitory concentration (MIC) of each strain was calculated by the percent growth across the plate multiplied by the antimicrobial concentration used in the plate. One-Way ANOVA with Tukey’s multiple comparison post-test or with Dunnet’s post-hoc test, as indicated, was used to calculate the level of significance for the tested strains.

### Purification of VexR

Proteins for the gel mobility shift assay were purified as follows. *E*. *coli* ER2566 carrying pMAL-c2 or pMAL-c2-*vexR* were grown overnight at 37°C with aeration. The cultures were then diluted 100-fold into LB broth with Cb and incubated at 37°C with shaking until reaching an OD_600_ of ~0.5 when isopropyl β-D-1-thiogalactopyranoside (IPTG) was added to a final concentration of 0.3 mM and the cultures were incubated for an additional 2 h. The cells were then harvested by centrifugation, the supernatant removed, and the pellet resuspended in column buffer (20 mM Tris-HCl, 200 mM NaCl, 1 mM EDTA) plus 1 mM phenylmethylsulfonyl fluoride (PMSF). The cells were then lysed using a M-11P Microfluidizer processor according to the manufacturer’s instructions (Microfluidics). The resulting lysates were cleared of particulate matter by centrifugation at 15,000 x g for 20 min at 4°C. The clarified supernatants (i.e. VexR-MBP or MBP) were diluted 1:6 with column buffer and loaded onto a 0.8 x 7 cm column containing 1 mL of amylose resin (New England Biolabs). The column was washed with 12 column volumes of column buffer before the bound proteins were eluted with elution buffer (20 mM Tris-HCl, 200 mM NaCl, 1 mM EDTA, 10 mM maltose). Protein concentrations were determined using the Coomassie Plus Assay kit according to manufacturer’s instructions (Thermo Scientific). The purity of the eluted proteins were assessed by SDS–PAGE with Coomassie Brilliant Blue R-250 staining.

### Electrophoretic Mobility Shift Assay (EMSA)

DNA fragments designated vexR1 (the nucleotide sequence between -129 and -46 of the *vexR* promoter region) and vexR2 (-59 to +21 of the *vexR* promoter region) were amplified from N16961 using the primers *vexR*-F1/*vexR*-R1 and *vexR*-F2/*vexR*-R2 primers, respectively. The fragments were then used as a template for a second PCR reaction with the flanking biotin primer. The biotin primer was biotinylated by the manufacturer (IDT) to end label the fragments. The resulting biotinylated probes (2.5 nM) were incubated with purified VexR-MBP or MBP in amounts ranging from 0 to 250 nM in 10 μl of binding buffer containing 10 mM Tris (pH 7.4), 150 mM KCl, 0.1 mM DTT, 0.1 mM EDTA (pH 8), and 200 μg/mL sheared salmon sperm DNA. The binding reactions were incubated at room temperature for 20 min before being subjected to electrophoresis on a nondenaturing 5% polyacrylamide TBE gel in 0.25x TBE buffer at 200V for 45 min. The DNA in the gel was transferred to a nylon membrane in 0.5x TBE buffer at 380 mA for 1 h, UV cross-linked, before the biotinylated probes were detected using the Chemiluminescent Nucleic Acid Detection Module (Thermo Scientific) and documented using a FluorChem E imaging system (Protein Simple).

## Results

### Genetic organization of the *vexRAB* operon

**Fig 1:**
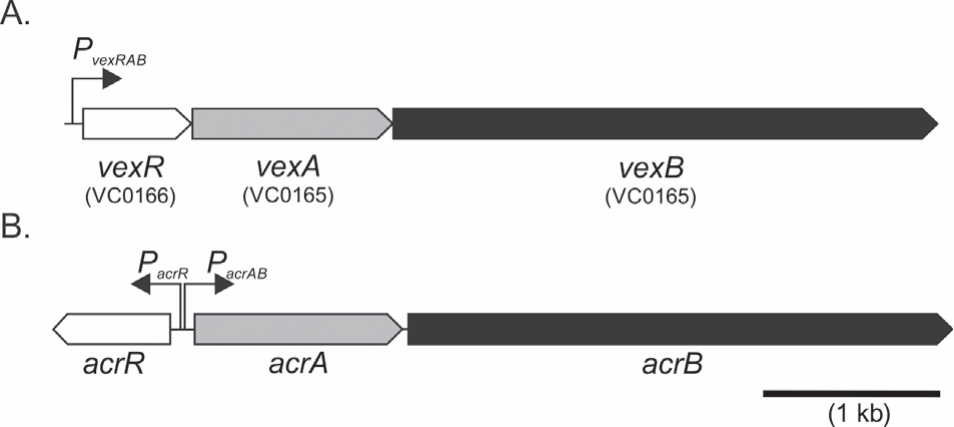
Genetic organization of RND efflux systems. (A) Schematic of the *V*. *cholerae vexRAB* operon. (B) Schematic of the *E*. *coli acrR-acrAB* locus. Genes encoding a TetR-family (white), membrane fusion family (grey), and RND-family (black) proteins are shown. Putative promoters for each respective operon are indicated by the thin black arrows.

In many gram negative bacteria the RND efflux systems encode a linked TetR family regulatory protein that functions as a repressor of the linked RND efflux system [27,28]. The *V*. *cholerae* genome encodes six RND efflux systems; among these six, only the VexAB RND system contained a linked TetR-family regulator which was previously named *vexR* (VC0166) [4,22]. The *vexR* gene was present as the first gene in the *vexRAB* operon (Fig. 1A). Relative to most other RND efflux systems, the genetic arrangement of *vexR* within the *vexRAB* operon is unusual [27–29]. In most RND efflux systems that encode a linked TetR regulator, the regulatory gene is expressed from a divergently expressed promoter that is upstream of and overlaps with the RND efflux system promoter. This arrangement is demonstrated by the archetypical *acrAB* RND efflux system in *E*. *coli* where *acrR* is encoded upstream and divergently from the *acrAB* efflux system (Fig. 1B) [28]. The AcrR protein functions to regulate the expression of the *acrAB* RND system in response to its antimicrobial substrates while also regulating its own expression [38].

### Expression of *vexRAB* is induced in response to VexAB efflux substrates

**Fig 2:**
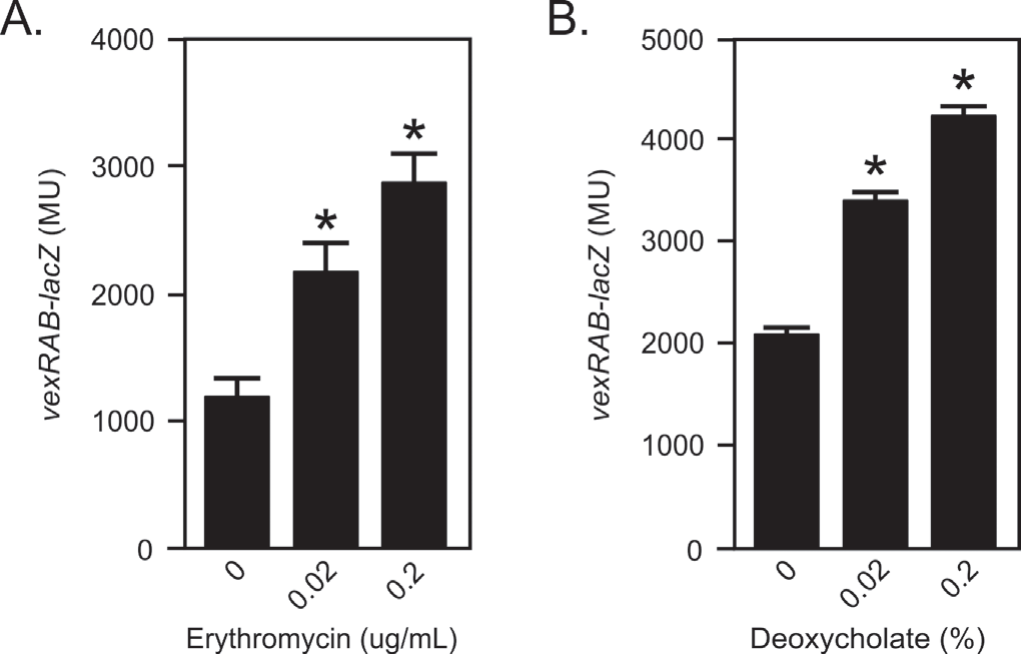
VexAB efflux substrates affect *vexRAB* expression. *V*. *cholerae* strain JB58 (Δ*lacZ* Sm^R^) containing pXB233 (*vexRAB*-*lacZ)* was grown under AKI conditions for 5 h with the indicated concentrations of (A) erythromycin or (B) deoxycholate when *vexRAB* expression was quantified as described in the methods. The reported data are in Miller Units (MU) and are the mean ± SD of three independent experiments. Statistical significance was determined relative to the media control by one-way ANOVA with Dunnet’s post-hoc test. * = P<0.05.

The role of the RND efflux pumps is to export their substrates out of the cell. As such, their expression is typically regulated by a feedback loop in response to the presence of their efflux substrates [28]. Since bile salts were shown to be major substrates of VexAB [4,5], we hypothesized that *vexRAB* expression may be regulated in response to bile salts. To test this we cultured *V*. *cholerae* JB58 (Δ*lacZ*) under virulence gene inducing conditions in AKI broth containing sub-lethal concentrations of deoxycholate and measured *vexRAB* expression using a *vexRAB-lacZ* reporter (i.e. pXB233). AKI growth conditions were selected because previous studies had indicated a linkage between RND efflux and virulence gene expression [5]. We also tested erythromycin, an antibiotic that is a substrate of VexAB. The results showed a concentration-dependent induction of *vexRAB* expression in response to both erythromycin (Fig. 2A) and deoxycholate (Fig. 2B). Deoxycholate at 0.2% resulted in a 2-fold increase in *vexRAB* expression while the presence of erythromycin at 0.2 μg/mL resulted in a nearly 3-fold increase in *vexRAB* expression. Based on these results we concluded that the *vexRAB* operon is regulated in response to deoxycholate and erythromycin, two of its known efflux substrates.

### VexR is required for expression of the *vexRAB* operon

**Fig 3:**
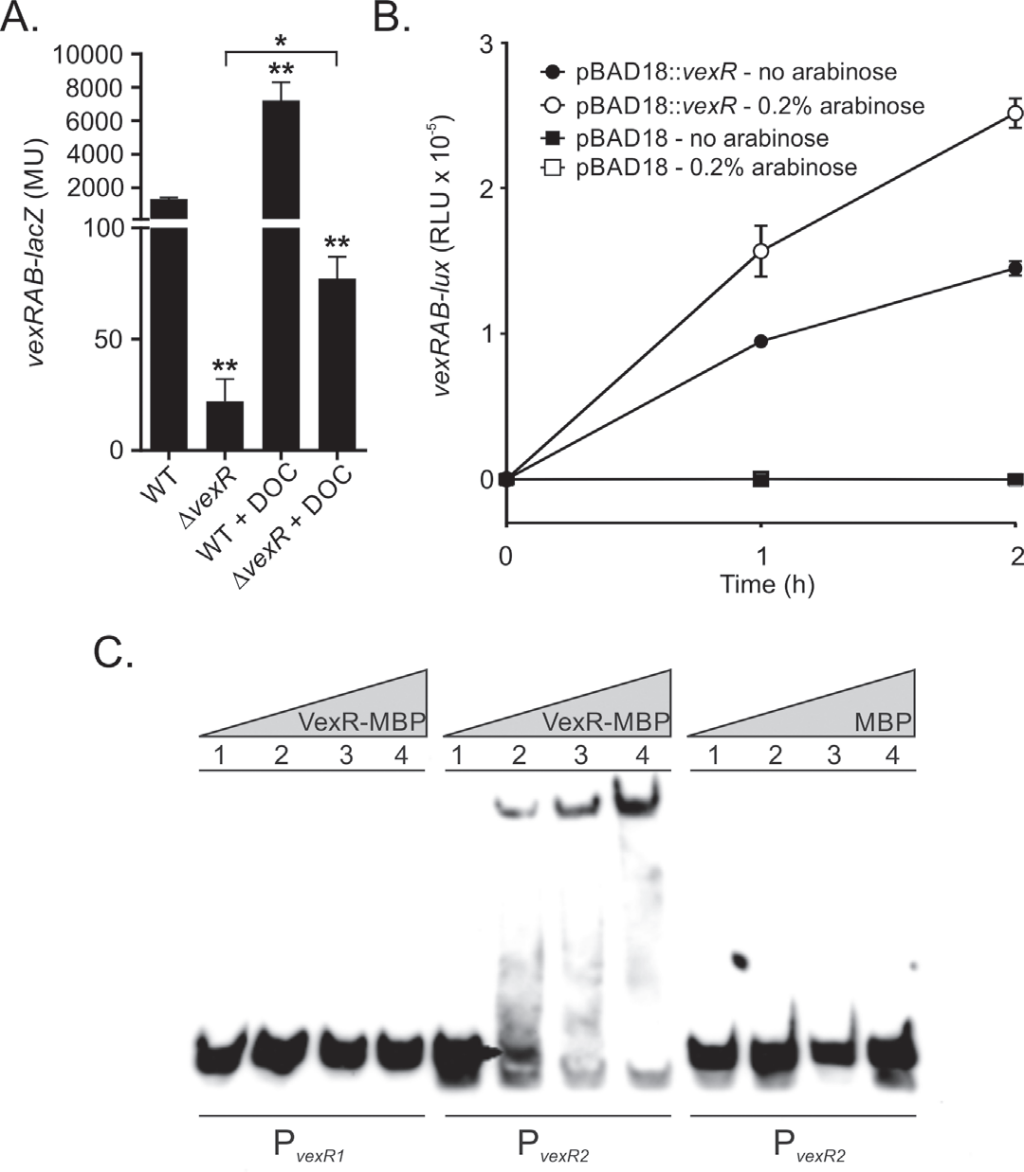
VexR is required for *vexRAB* expression. (A) N16961 WT and Δ*vexR* containing pXB233 (*vexRAB-lacZ)* were grown in LB broth. At 3.5h deoxycholate (DOC) was added to a final concentration of 0.2% and the cultures were incubated for an additional 30 min before *vexRAB* expression was determined as described in the methods. Data is the mean of three independent experiments ± SD. Significance was determine by one-way ANOVA with Tukey-Kramer multiple comparison test. Unless otherwise indicated, asterisks are significance relative to WT (*p<0.05; **p<0.001). (B) *E*. *coli* containing pDT1076(*vexRAB-lux*) and either pJB703(pBAD18::*vexR*) or pBAD18 was grown in LB broth with or without 0.2% arabinose and bioluminescence was assayed at 0h, 1h, and 2h. Error bars indicate the mean ± SD of three replicates. The results are representative of three independent experiments. (C) Gel mobility shift assay showing the binding of VexR to the *vexRAB* promoter. The promoter was split into two fragments (*vexR1*) -129 to -46 and (*vexR2*) -59 to +21 relative to the ATG start site. Biotin labeled DNA (2.5nM) from *vexR1* (lane 1–4) or *vexR2* (lane 5–12) fragments was incubated with either purified VexR-MBP or MBP as indicated at 0 nM (lane 1), 25 nM (lane 2), 100 nM (lane 3), or 250 nM (lane 4) prior to electrophoresis. Specific binding reaction, detection, and visualization are discussed in the Material and Methods.

The finding that *vexRAB* expression was induced in response to VexAB efflux substrates indicated the possibility that the *vexRAB* system was regulated in a fashion that was similar to other RND efflux systems. Given that most TetR-family regulators function as repressors in the absence of their efflux substrates, we hypothesized that VexR would function to repress *vexRAB* expression. If this was true, then deletion of *vexR* should result in increased *vexRAB* expression. We therefore compared *vexRAB-lacZ* expression in JB58 and an isogenic Δ*vexR* mutant strain. The strains were grown in LB broth to an OD_600_ of 0.8 when *vexRAB-lacZ* expression was quantified. The results showed that *vexRAB* expression was significantly decreased in the Δ*vexR* strain relative to WT (Fig. 3A). This finding suggested that VexR was a positive, rather than a negative regulator of the *vexRAB* operon.

We examined whether the substrate-dependent induction is contingent on the presence of *vexR* because *vexRAB* expression was increased in response to the VexAB substrates (Fig. 2). We chose to focus on deoxycholate as an inducer because it is the biologically most relevant *vexAB* substrate with regards to *V*. *cholerae* pathogenesis [5]. Growth of WT in the presence of 0.2% deoxycholate resulted in a ~5.5-fold increase in *vexRAB-lacZ* expression (Fig. 3A). Deletion of *vexR* greatly reduced the deoxycholate-dependent induction of *vexRAB* expression. This result further supported the conclusion that *vexR* was required for *vexRAB* expression. While VexR appeared to be required for robust *vexRAB* expression, deoxycholate still induced *vexRAB* expression by ~2-fold in the Δ*vexR* strain relative to the LB broth control, although the expression level was greatly reduced compared to JB58 grown in LB broth (Fig. 3A). This suggested that there may be other factors involved in regulating *vexRAB* expression; a scenario similar to the *E*. *coli acrAB* operon where multiple factors contribute to its expression. Taken together these findings show that *vexR* contributes to the positive regulation of *vexRAB*.

The VexAB RND efflux system is the only RND efflux system that encodes a linked regulatory system. While the VexCD RND efflux system has been shown to be under negative control by BreR [6], the regulatory mechanisms controlling the expression of the other five RND efflux systems were unknown. We therefore tested the hypothesis that VexR may regulate the expression of the other five RND efflux systems. We introduced plasmid based *lacZ* reporters for each of the other five RND efflux systems (i.e. *vexCD*, *vexEF*, *vexGH*, *vexIJK*, and *vexLM*) into JB58 and its isogenic *vexR* deletion strain. The resulting strains were then cultured in LB broth in the presence or absence of 0.02% deoxycholate for two hours when β-galactosidase activity was quantified. The results showed that there was no significant difference in the expression of the five tested RND efflux systems in WT relative to the Δ*vexR* mutant under either growth condition (S1 Fig.). From this we concluded that *vexR* was specific for the *vexRAB* operon.

To further confirm that VexR contributes to activation of the *vexRAB* operon we tested whether recombinant *vexR* expression would activate transcription of the *vexRAB* promoter in *E*. *coli*. We therefore transformed *E*. *coli* bearing pDT1076 (*vexRAB-lux*) with pBAD18 (empty vector control) or pBAD18::*vexR*. The resulting strains were then grown in LB broth plus or minus 0.2% arabinose (to induce *vexR* expression from the arabinose inducible promoter in pBAD18) and luminescence production was measured at zero, one and two hours. The results showed that the empty vector control (i.e. pBAD18) did not affect *vexRAB* expression (Fig. 3B). This indicated that neither pBAD18, nor arabinose, affected the expression of the *vexRAB-lux* reporter. In contrast, there was a marked increase in luminescence production in the strain containing pBAD18::*vexR*. The presence of pBAD18::*vexR*, even in the absence of arabinose, was sufficient to induce *vexRAB* expression as evidenced by the increase in luminescence production at one and two hours (Fig. 3B); this may reflect leaky *vexR* expression from pBAD18::*vexR*. The addition of arabinose to the broth further increased *vexRAB* expression. Based on these results, plus the results presented in Fig. 3A, we concluded that *vexR* contributes to positive regulation of the *vexRAB* operon.

### VexR binds to the *vexRAB* promoter directly

The above experiments suggested that VexR may act directly at the *vexRAB* promoter. To test this hypothesis we performed gel mobility shift assays using VexR-MBP with the *vexRAB* promoter. The 5’ upstream region of *vexRAB* was arbitrarily split into two fragments that covered from -129 to -46 (*vexR1*) and -59 to +21 (*vexR2*) relative to the ATG start site (Fig. 3C). The results of the gel shift assays showed that purified VexR-MBP was able to shift the *vexR2* promoter fragment, but not the *vexR1* promoter fragment. Incubation of the *vexR2* fragment with MBP did not result in a shift confirming that the results were due to VexR (Fig. 3C). These results validated that VexR directly binds to the *vexRAB* promoter and supports the conclusion that VexR directly regulates *vexRAB* expression.

### VexR contributes to antimicrobial resistance

If VexR contributes to positive regulation of the *vexRAB* operon, we hypothesized that the deletion of *vexR* should increase *V*. *cholerae* susceptibility to antimicrobial compounds that are substrates of VexAB. To test this we determined the MIC of deoxycholate, erythromycin, and Triton X-100 for WT strain JB58 and its isogenic Δ*vexR* mutant. While deoxycholate, erythromycin, and Triton X-100 are all substrates of VexAB, other RND efflux systems possess redundant efflux activity for deoxycholate and Triton X-100. The results showed a 2.7-fold decrease in the erythromycin MIC in the Δ*vexR* mutant (Table 2). In contrast, there was no change in the susceptibility of the Δ*vexR* mutant to deoxycholate or Triton X-100. These results contrast the resistance profile of a Δ*vexB* mutant. For example, the erythromycin MIC for the *vexR* mutant was 1.65 μg/mL compared to 0.07 μg/mL for the *vexB* mutant. Likewise, the Triton X-100 MIC for the *vexR* mutant was >3% while the *vexB* mutant MIC was 0.0017%. This indicated that the *vexR* mutant exhibited a phenotype that was intermediate between WT and the Δ*vexB* strain. Since VexB is the only RND efflux pump that contributes to erythromycin resistance [4], these results suggest that *vexAB* is expressed at a low basal level in the Δ*vexR* mutant.

**Table 2:** Antimicrobial susceptibility of *vexR* mutants.

		**MIC (s.d.)^1^**		
**Strain**	**Genotype**	**Em (μg/mL)**	**Doc (%)**	**TX-100 (%)**
JB58	WT	4.40 (2.1)	>3 (0)	>3 (0)
XBV218	Δ*vexR*	1.65 (0.9)^2^ ^,^ ^3^	>3 (0)	>3 (0)
JB495	Δ*vexB*	0.07 (0.005)^2^	>3 (0)	0.0017 (0.0011)^2^
JB692	Δ*vexD*	ND	>3 (0)	ND
JB718	Δ*vexR*Δ*vexD*	ND	0.020 (0.001)^2^ ^,^ ^4^	ND
JB694	Δ*vexB*Δ*vexD*	ND	0.007 (0.003)^2^	ND

^(1)^Minimum Inhibitory Concentration (MIC) for erythromycin (Em), deoxycholate (Doc), and Triton X-100 (TX-100) for the indicated N16961 strains with standard deviations in parenthesis.

^(2)^ P< 0.05 relative to WT.

^(3)^ P<0.05 relative to Δ*vexB*.

^(4)^ P<0.05 relative to Δ*vexBD*. ND = not determined.

The fact that *vexR* deletion did not affect deoxycholate resistance was expected as previous studies have shown that the VexCD RND efflux system was redundant with VexAB for bile salt resistance [4]. Because of overlapping specificity for deoxycholate, mutation of both *vexB* and *vexD* are required to produce a bile salt hypersensitive phenotype [4]. Therefore, to address whether *vexR* contributed to deoxycholate resistance we examined the effect of *vexR* deletion in a Δ*vexD* background. The results showed that deletion of *vexR* in a Δ*vexD* background reduced the deoxycholate MIC to 0.019%; a MIC that was 2.7-fold greater than the deoxycholate MIC observed with the Δ*vexB*Δ*vexD* mutant (Table 2). The intermediate resistance phenotype of the Δ*vexR*Δ*vexD* mutant (relative to the Δ*vexB*Δ*vexD* mutant) further supported the conclusion that the *vexRAB* efflux system is expressed at a low basal level in the *vexR* mutant.

VexAB is the primary RND efflux system involved in Triton X-100 resistance [4]. Consistent with this, a >1,700-fold increase in Triton X-100 susceptibility was observed in a Δ*vexB* mutant (Table 2). However, deletion of *vexR* did not affect Triton X-100 susceptibility (Table 2). This suggests that the low-level *vexAB* expression in the *vexR* mutant was sufficient to provide WT-level Triton X-100 resistance. Alternatively, it is possible that deletion of *vexR* resulted in the induction of other resistance traits that contributed to Triton X-100 resistance and thus compensated for the reduction in *vexRAB* expression in the *vexR* mutant.

The collective MIC data indicated that VexR was required for maximum resistance to erythromycin and deoxycholate under the tested conditions. When *vexR* was deleted, the contribution of VexAB to antimicrobial resistance was significantly reduced, but not to a level that was equal to a *vexB* null mutant. This suggested that *vexAB* was expressed at a low basal level in the Δ*vexR* mutant.

We recently reported that activation of the Cpx membrane stress response system can enhance *vexRAB* expression in *V*. *cholerae* [26]. We also showed that *vexB* deletion resulted in activation of the *V*. *cholerae* Cpx system which suggested that *vexRAB* and the Cpx system were reciprocally regulated. Based on this we hypothesize that if *vexR* was required for *vexAB* expression, then mutation of *vexR* should also result in activation of the Cpx system. To test this hypothesis we assayed *cpxP* expression. The expression of *cpxP* is regulated by CpxR and has been used as a reporter for the activation state of the cpx system [26,37]. We introduced a *cpxP-lacZ* chromosomal reporter into Δ*vexR* and Δ*vexR*Δ*cpxR* mutants. We then compared *cpxP-lacZ* expression on LB-X-gal agar plates. In these assays we also included WT, Δ*vexB*, Δ*vexBDFHKM* and Δ*cpxR* mutants as controls. The results showed that WT produced white colonies on LB agar (S2 Fig.). This indicated that the Cpx system was inactive under standard growth conditions as previously reported [37]. Growth of the same strain in the presence of CuCl_2_, which is an inducer of the Cpx system, resulted in the formation of dark blue colonies. This confirmed that the Cpx reporter system was functioning as expected. In contrast to WT, the Δ*vexR* mutant produced light blue colonies on LB agar that were similar in appearance to the Δ*vexB* mutant. This indicated that deletion of *vexR*, like deletion of *vexB*, resulted in activation of the Cpx system.

Since deletion of *vexR* activated the Cpx system, we tested whether the Cpx system affected *vexRAB* expression in the Δ*vexR* background. If this was true we predicted that deletion of *cpxR* in the Δ*vexR* background would result in decreased *vexRAB* expression and increased sensitivity to VexAB efflux substrates. We first determined the effect of individual and combined deletions of *vexR* and *cpxR* on erythromycin resistance. The results showed that the erythromycin MIC for the Δ*vexR*Δ*cpxR* mutant was not different from the Δ*vexR* mutant (S2 Table) suggesting that the Cpx system is not contributing to enhance resistance to VexAB substrates in the *vexR* mutant. Consistent with this result, deletion of *cpxR* in a Δ*vexR* mutant did not affect *vexRAB* expression (S3 Fig.). Taken together these results confirmed that *vexR* deletion activated the Cpx system, but that the Cpx system did not affect the basal expression of *vexRAB* in the absence of *vexR*. One potential explanation for this observation is that CpxR-dependent enhancement of *vexRAB* expression may require *vexR*.

### Overexpression of *vexR* enhances resistance to deoxycholate

We hypothesized that if *vexR* was required for *vexRAB* upregulation and antimicrobial resistance, then *vexR* overexpression should complement a Δ*vexR* mutant for growth in the presence of deoxycholate and erythromycin. We tested this hypothesis by determining the deoxycholate and erythromycin MIC for WT, Δ*vexR* and Δ*vexR*Δ*vexD* strains that expressed *vexR* from the arabinose promoter in pBAD18. In these experiments the respective strains containing pBAD18 or pBAD18::*vexR* were grown on antimicrobial gradient agar plates that contained a range of different arabinose concentrations. However, we were unable to identify an arabinose concentration which resulted in complementation of the tested mutants for deoxycholate or erythromycin resistance (data not shown). We considered that this result could have been an artifact of the pBAD18 expression system, and therefore we cloned *vexR* into the low copy number IPTG-inducible pMMB66EH expression vector and repeated the complementation experiments and obtained identical results.

The reason that we were unable to complement the *vexR* mutant is not clear. The inability to complement the *vexR* deletion likely did not result from the introduction of a secondary mutation during the construction of the *vexR* mutant since DNA sequencing confirmed the integrity of the *vexR* deletion construct (i.e. pWM91::Δ*vexR*) and the *vexRAB* locus in the *vexR* deletion strain. Further, the Δ*vexR* and Δ*vexR*Δ*vexD* mutants were independently created from different parental strains, making it unlikely that the complementation defect was due to an unlinked spontaneous mutation. The lack of complementation was also not due to mutations introduced into the complementing plasmids as we confirmed the DNA sequence of the complementing plasmids. This latter conclusion is further supported by the observation that the presence of pBAD18::*vexR* activated *vexRAB-lacZ* expression in *E*. *coli* (Fig. 3B).

**Fig 4:**
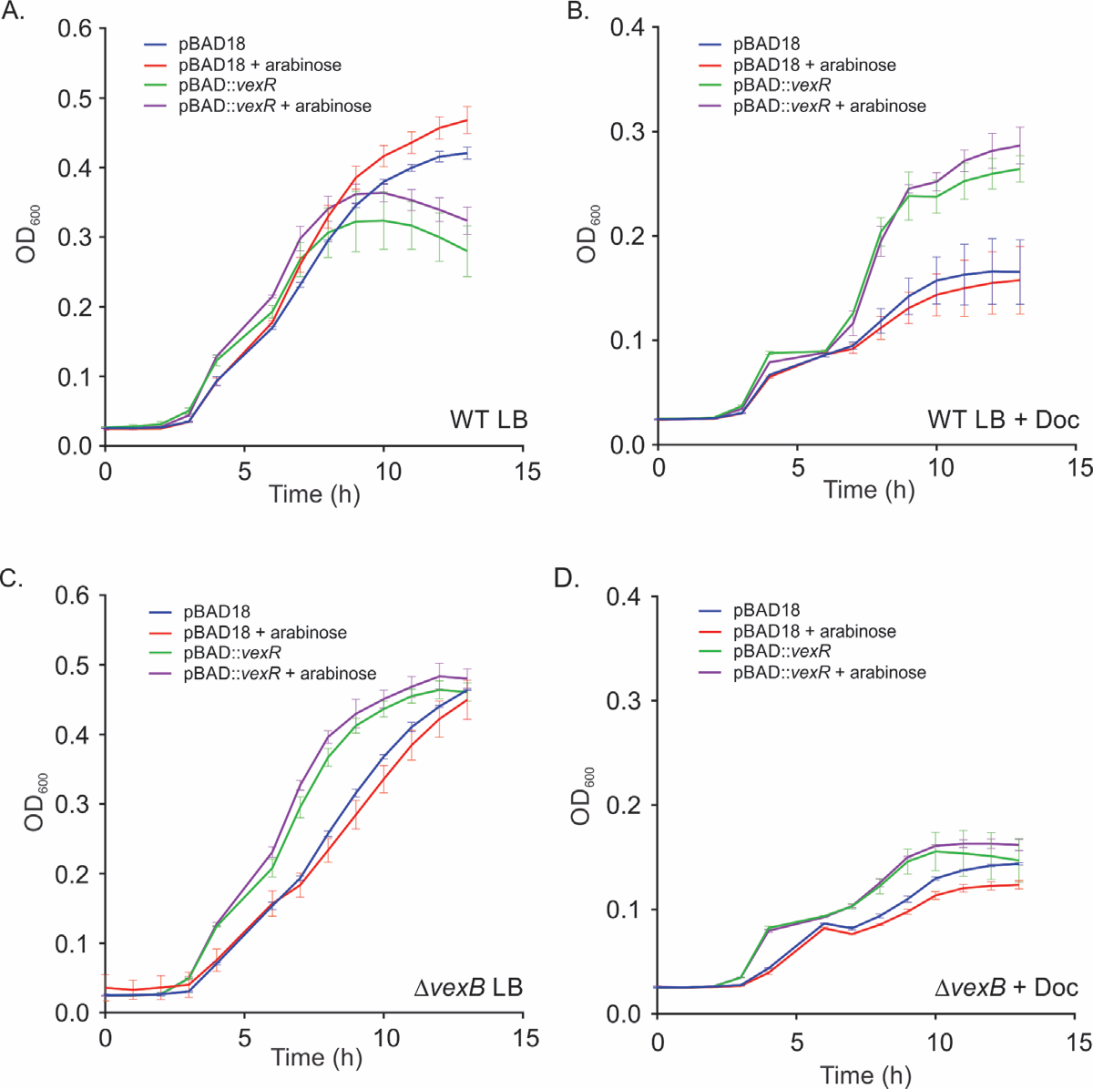
VexR contributes to *V*. ***cholerae* survival under inhibitory antimicrobial conditions.** *V*. *cholerae* N16961 WT (A & B) and Δ*vexB* (C & D) containing pJB703(pBAD18::*vexR*) or pBAD18 were grown in triplicate wells of microtiter plates containing LB broth (A & C) or LB broth plus 0.015% deoxycholate (B & D). Expression of *vexR* was induced by adding 0.1% arabinose to the growth media as indicated. Cell growth was monitored as the change in the optical density at 600 nm and plotted versus time as the mean ±SEM. The results are representative of three independent experiments.

Since the *vexR* deletion mutant could not be complemented by ectopic *vexR* expression, we sought another method to confirm that VexR contributed to antimicrobial resistance. We hypothesized that if *vexR* was indeed an activator of the *vexRAB* operon, then episomal expression of *vexR* in WT should result in enhanced growth in the presence of sub-lethal concentrations of VexAB substrates. To test this hypothesis we generated growth curves for WT(pBAD18::*vexR*) and WT(pBAD18) in LB broth containing a sub-lethal concentration of deoxycholate. The results of this analysis showed that in the absence of deoxycholate, WT(pBAD18::*vexR)* grew equally well during logarithmic phase of growth as WT(pBAD18) through six hours (Fig. 4A). Thereafter WT(pBAD18) growth continued to increase whereas the strain bearing pBAD18::*vexR* peaked and the cell density declined through 12 hours. This indicated that over-expression of *vexR* is detrimental under standard laboratory growth conditions. We speculated that this was a result of increased *vexAB* expression which has been suggested to be growth inhibitory under non-selective conditions [27,39]. When the same strains were cultured in LB broth containing deoxycholate, WT (pBAD18::*vexR*) exhibited a growth advantage over the empty vector control regardless of the presence or absence of arabinose (Fig. 4B). The addition of arabinose did not significantly affect the growth of WT(pBAD18::*vexR)* relative to the same culture grown in the absence of arabinose (Fig. 4B). The complementation results were reminiscent of the findings observed for *vexR* activation of *vexRAB* in *E*. *coli* (Fig. 3B) where *vexR* enhanced *vexRAB-lux* expression even in the absence of arabinose, and further enhanced *vexRAB-lux* expression with the addition of arabinose. Taken together these results provide additional support for the hypothesis that *vexR* contributes to *vexRAB* activation.

The above results suggested that episomal *vexR* expression enhanced deoxycholate resistance, but did not allow us to discriminate if the resistance phenotype was mediated by VexAB or some other factor. If enhanced growth was due to VexR activation of VexAB production, then deletion of *vexB* should alleviate the *vexR*-dependent growth enhancement in the presence of deoxycholate. We therefore repeated the above growth experiments in a *vexB* deletion strain. The results showed that the growth of the Δ*vexB*(pBAD18) control strain was slightly attenuated relative to Δ*vexB*(pBAD18::*vexR*) in LB broth (Fig. 4C). This suggested that *vexR* may impart a growth advantage in the *vexB* mutant during growth in LB broth. The growth of the Δ*vexB* strain was significantly inhibited in the presence of deoxycholate (Fig. 4D). Unlike what was observed in the WT background, *vexR* expression in the Δ*vexB* mutant did not significantly affect cell growth (Fig. 4D). This suggested that the enhanced growth observed with *vexR* in WT cells grown in the presence of deoxycholate may have resulted from *vexR*-dependent activation of the *vexRAB* operon (Fig. 4B). We also tested if pBAD18::*vexR* would provide a growth advantage to a *vexR* deletion strain under the same growth conditions, but the results were congruent with the gradient agar plate results and showed that pBAD18::*vexR* did not complement the Δ*vexR* mutant (S4 Fig.). Taken together the collective results are consistent with the idea that *vexR* overexpression enhanced *V*. *cholerae* growth in the presence of deoxycholate via the VexAB RND efflux system.

### Deletion of the RND efflux systems induces *vexRAB* expression

**Fig 5:**
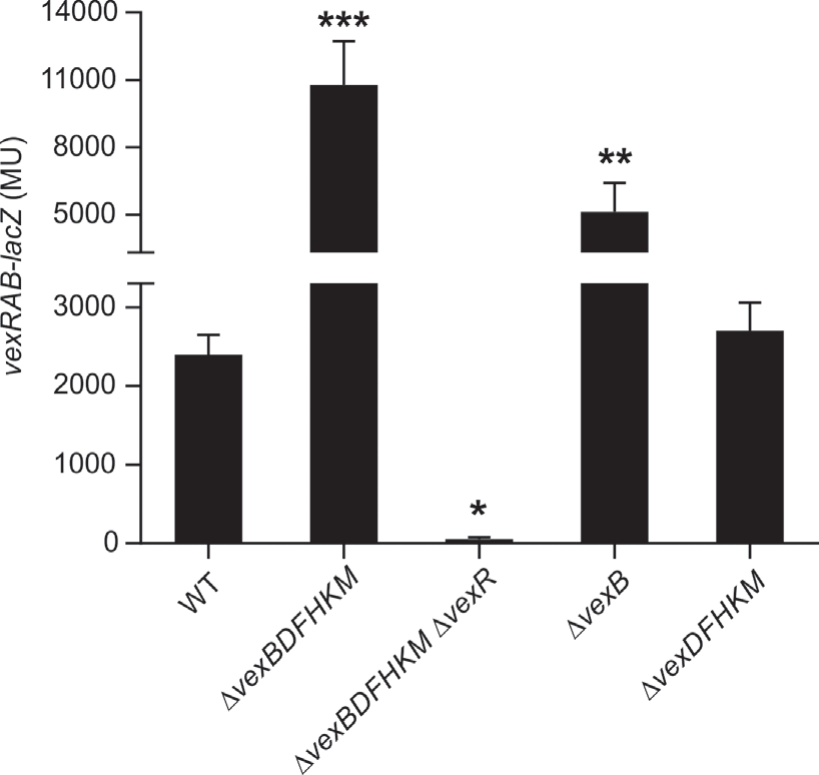
Expression of *vexRAB* is induced in *V*. ***cholerae* RND efflux mutants.** The indicated N16961 strains containing pXB233 (*vexRAB-lacZ*) were grown for 5 h under AKI conditions when *vexRAB* expression was quantified as described in the methods. Strain JB485 is RND negative and lacks all six RND efflux pump proteins. Error bars indicate ± SD of three independent experiments. One-way ANOVA with Dunnet’s post-hoc test was used to determine significant differences relative to WT. * = P<0.05; ** = P<0.01; ***P = <0.0001.

Recent studies in *E*. *coli* suggested that the RND systems may have evolved to remove potentially toxic metabolites from the cell cytoplasm [40–42]. If this was true, we hypothesized that *vexRAB* expression would be upregulated in RND negative cells due to the accumulation of metabolites that were substrates of the RND efflux systems. Consistent with this hypothesis *vexRAB* has been reported to be upregulated in RND negative strain JB485 [26]. To further expand upon this and to determine whether *vexR* contributed to this phenotype we introduced the *vexRAB-lacZ* reporter into WT, RND deficient strain JB485, and JB485Δ*vexR*. The resulting strains were then cultured under AKI conditions in AKI broth for 5 h when *vexRAB* expression was assessed (Fig. 5). AKI growth conditions were selected for these studies because previous work had indicated a linkage between RND efflux and virulence gene expression [5]. The results showed a ~4.5-fold increase in *vexRAB* expression in JB485 confirming previous results [26]. Deletion of *vexR* in JB485 reduced *vexRAB* expression to near background levels which suggested that the increase in *vexRAB* expression in JB485 was dependent upon *vexR* (Fig. 5).

We next tested whether increased *vexRAB* expression in JB485 was due to loss of *vexB* or a result of the deletion of all six RND efflux systems. We first quantified *vexRAB* expression in a Δ*vexB* mutant. The results showed that *vexRAB* expression increased in the *vexB* mutant (~3-fold), but to a level that was less than observed in JB485 (Fig. 5). This suggested that at least one of the other RND systems can partially compensate for the loss of *vexB*. The expression of *vexRAB* in a Δ*vexDFHKM* (*vexB*+) was not significantly different from WT. This latter finding was consistent with previous work showing that *vexB*, due to its broad substrate specificity, was able to complement for the loss of the other five RND efflux systems [5,24]. Taken together these results indicated that *vexRAB* expression was responsive to the RND efflux status of the cell and that deletion of *vexB* resulted in *vexRAB* expression being elevated. The higher level of *vexRAB* expression in JB485 relative to the *vexB* mutant suggested that one or more of the other RND efflux systems were able to efflux the inducing factor(s). Based on these results we inferred that *V*. *cholerae* compensates for reductions in RND efflux activity by upregulating *vexRAB* expression. Exactly how *V*. *cholerae* senses efflux activity was unclear, but we speculated this resulted from the intracellular accumulation of natural substrates of the RND efflux systems in the absence of RND efflux activity.

### The expression of *vexRAB* is altered in metabolic mutants

We tested whether *V*. *cholerae* upregulated *vexRAB* expression in response to the accumulation of metabolic byproducts. Our approach for these experiments was based on the assumption that the mutation of metabolic genes would disrupt biosynthetic pathways and result in the intracellular accumulation of chemical intermediates of the targeted biochemical pathway which would then activate *vexRAB* expression. To conduct these studies we obtained a number of metabolic mutants from a defined transposon mutant library that was constructed in *V*. *cholerae* El Tor strain C6706 [33]. We selected mutants that targeted a number of different metabolic pathways including some that have been shown to affect *E*. *coli acrAB* expression [42]. This included mutants that affected purine metabolism, cysteine metabolism, sulfur metabolism, vibriobactin biosynthesis, glycolysis, gluconeogenesis, pyruvate metabolism, amino acid metabolism, and the citric acid cycle (S1 Table).

The metabolic mutants were transformed with pDT1777 (*vexRAB-lux*) and cultured in AKI broth for five hours when *vexRAB* expression was quantified. While we were most interested in the mutants that activated *vexRAB* expression, we noted several mutations reduced *vexRAB* expression. Based on the idea that *vexRAB* expression is modulated by metabolite accumulation, one explanation for reduced *vexRAB* expression is the loss of down-stream activators. However, we cannot exclude that the observed reduction of *vexRAB* expression was an artifact resulting from cellular byproducts inhibiting the luciferase reporter [43]. Three mutants were identified (VC1172, VC1579, and VCA1046) that resulted in a >2-fold increase in *vexRAB* expression (S1 Table). VC1172 encodes TrpD which functions in tryptophan biosynthesis; VC1579 encodes AlmE which is a lipid A modification enzyme [44]; and VCA1046 encodes mannitol-1-phosphate 5-dehydrogenase and is involved in mannitol metabolism. These results confirmed that *vexRAB* expression was upregulated in response to interruption of at least three metabolic pathways. To further investigate this phenomenon we focused on tryptophan biosynthesis due to the availability of mutants and two chemical intermediates in the tryptophan biosynthetic pathway.

### Disruption of the tryptophan biosynthetic pathway affects *vexRAB* expression

**Fig 6:**
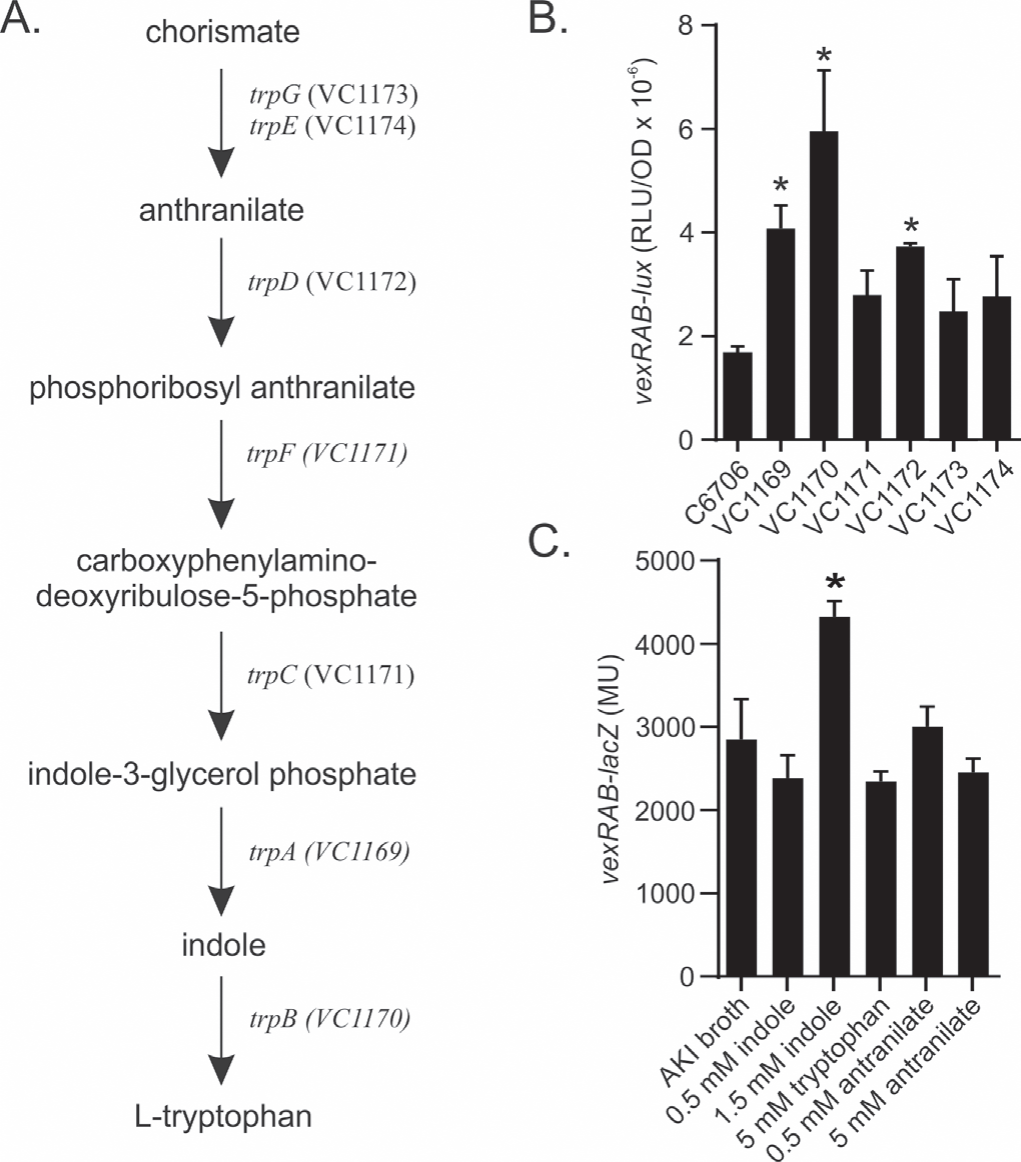
Indole activates *vexRAB* expression. (A) Schematic of the *V*. *cholerae* tryptophan biosynthetic pathway. (B) The indicated C6706 strains bearing pDT1777 (*vexRAB-lux*) were grown in 96-well plates under AKI conditions for 5 h when luminescence (RLU) and OD_600_ were measured. Data are the mean +/- SEM of three independent assays. Two-way Anova with Dunnet’s post-hoc test compared to WT was used to determine significance. * = P<0.01; ** = P<0.0001. (C) *V*. *cholerae* N16961 containing pXB233 (*vexRAB-lacZ*) was grown with or without indole, L-tryptophan, or anthranilic acid at the indicated concentrations under AKI conditions for 5 h before *vexRAB* expression was determined as described in the methods. Bars indicate the mean +/- SD of three independent experiments. One-way ANOVA with Dunnet’s post-hoc test was used to determine significant changes relative to growth in media alone. * = P<0.001.

Mutation of *trpD* (VC1172) resulted in the strongest induction of *vexRAB* expression suggesting that tryptophan biosynthesis intermediates may function as inducers of the *vexRAB* operon. To investigate this we examined *vexRAB* expression in six different tryptophan biosynthetic transposon mutants (Fig. 6A). The results showed that *vexRAB* expression increased 3-fold in the *trpB* mutant, and ~2-fold in the *trpA* and *trpD* mutants (Fig. 6B). Mutation of *trpB* (VC1170) is predicted to result in indole accumulation while mutation of *trpA* (VC1169) and *trpD* (VC1172) would result in indole-3-glycerol phosphate and anthranilate accumulation, respectively. This suggests that indole, indole-3-glycerol phosphate and anthranilate could contribute to *vexRAB* induction.

If intermediates of tryptophan biosynthesis were *vexRAB* inducers, we hypothesized that adding these compounds to the growth media would activate *vexRAB* expression. As anthranilate and indole were available from commercial sources, we tested if the addition of these compounds affected *vexRAB-lacZ* expression in WT. The results showed that indole at 1.5 mM increased *vexRAB* expression by ~1.5 fold (Fig. 6C). This finding, combined with the observation that *vexRAB* was induced in the *trpB* mutant (Fig. 6B), suggested that indole may contribute to *vexRAB* activation. The addition of anthranilate or tryptophan did not have a significant effect on *vexRAB* expression (Fig. 6C). There are at least two explanations for this result. First, it is possible that mutation of *trpD* may have pleiotropic effects on other metabolic pathways which could be responsible for the effects on *vexRAB* expression. Second, it is also possible that *V*. *cholerae* lacks an anthranilate transport system which would limit the effects of exogenous anthranilate.

### Indole is a substrate of the VexAB RND efflux system

Indole is an intermediate in the biosynthesis of tryptophan and is also produced by *V*. *cholerae* as a byproduct of tryptophan degradation. Indole has also been shown to function as a signaling molecule in biofilm formation [45,46]. At high concentrations indole is toxic, which suggested that it may be a potential substrate of the *V*. *cholerae* RND efflux systems. We therefore tested if the RND efflux systems contributed to indole resistance by comparing the growth of WT and the RND deficient strain JB485 (Δ*vexBDFHKM*) on indole gradient agar plates. The results showed that the WT MIC was 2.1 mM while the JB485 MIC was 1.6 mM (Table 3). This suggested that the *V*. *cholerae* RND efflux systems contributed to indole resistance. In an effort to determine which RND efflux systems contributed to indole resistance we examined mutants lacking each of the six RND efflux systems pumps (i.e. *vexB*, *vexD*, *vexF*, *vexH*, *vexK*, *vexM*). Only the *vexB* mutant had a small but significant decrease in the indole MIC (1.8 mM), which was higher than the MIC exhibited by JB485 (Table 3). Although small, the change in the *vexB* MIC was consistent with the idea that indole is a substrate of the VexAB RND efflux system. The discrepancy between the *vexB* and JB485 MICs suggest that at least one of the other RND efflux systems function in indole resistance. All together this data indicates that indole may be a substrate of the RND efflux systems and that indole may serve as an inducer of the *vexRAB* operon.

**Table 3:** Minimum inhibitory concentration of indole for RND mutants.

**Strain**	**Genotype**	**Indole MIC (s.d)^1^**
JB58	WT	2.1 (0.1)
XBV218	Δ*vexR*	2.0 (0.2)
JB495	Δ*vexB*	1.8 (0.2)^2^
JB692	Δ*vexD*	2.0 (0.2)
JB432	Δ*vexF*	2.0 (0.1)
JB116	Δ*vexH*	2.1 (0.1)
JB528	Δ*vexK*	2.1 (0.1)
JB114	Δ*vexM*	2.0 (0.1)
JB485	Δ*vexBDFHKM*	1.6 (0.1)^3^

^(1)^Minimum Inhibitory Concentration (MIC) of Indole (mM) for the indicated N16961 strains. One-way ANOVA with Dunnet’s post-hoc test was used to determine statistical difference relative to WT.

^(2)^ P< 0.05 relative to WT;

^(3)^ P<0.001 relative to WT.

## Discussion

The VexAB RND efflux system was the only *V*. *cholerae* RND system that was associated with a linked TetR family regulator (Fig. 1). In contrast to what is observed with most RND loci, VexR was encoded as the first gene in the *vexRAB* operon. VexR contributed to *vexRAB* expression as evidenced by the finding that *vexR* deletion decreased *vexRAB* expression (Fig. 3A) and resulted in increased susceptibility to the VexAB substrate erythromycin (Table 2). These results indicated that *vexR* contributed to the positive regulation of the *vexRAB* operon. This conclusion was supported by subsequent experiments showing that VexR directly bound the *vexRAB* promoter (Fig. 3C), that VexR was able to increase *vexRAB* expression in *E*. *coli* (Fig. 3B), and that *vexR* overexpression in *V*. *cholerae* resulted in a *vexB*-dependent growth advantage in the presence of sub-lethal concentrations of deoxycholate (Fig. 4).

The finding that VexR was required for *vexRAB* expression was unexpected. The vast majority of TetR-family regulators behave as transcriptional repressors [30]. Thus our findings highlight differences in the regulation of *vexRAB* relative to its orthologous Enterobacteriaceae system (i.e. *acrR-acrAB*). In *E*. *coli acrR* functioned as a negative regulator of *acrAB* while global regulatory systems such as the Mar operon functioned to positively regulate *acrAB* expression in response to antimicrobial exposure (Fig. 1) [28,29]. Although *V*. *cholerae* lacks the Mar operon, VexR could function in a similar role as the Mar operon by contributing to *vexRAB* expression in response to antimicrobial exposure. It is worth noting that the *vexRAB* and *vexGH* RND efflux systems and the Cpx system are reciprocally regulated in *V*. *cholerae* [26]. Recent studies showed that mutation of *vexB* or *vexH* resulted in activation of the Cpx system while activation of the Cpx system resulted in upregulation of *vexRAB* and *vexGH*. Herein our results show that deletion of *vexR* resulted in activation of the Cpx system (S2 Fig.), but that Cpx activation did not affect *vexRAB* expression (S3 Fig.) or VexAB mediated antimicrobial resistance (S2 Table). This result may suggest that the Cpx-dependent upregulation of the *vexRAB* operon is dependent on *vexR*. The fact that the Cpx system does not respond to antimicrobial substrates of the *V*. *cholerae* RND efflux systems further indicates that *vexRAB* expression is under the influence regulatory systems that respond to distinct stimuli. This could explain the inability to transcomplement the *vexR* deletion strain. It is possible that VexR-dependent regulation of the *vexRAB* operon is dependent on VexR interaction with other regulatory elements. Alternatively, cis-acting sequencing within the *vexR* open reading frame could be required for *vexRAB* upregulation.

The expression of *vexRAB* was highly elevated upon loss of RND-mediated efflux (Fig. 5). This upregulation was abolished when *vexR* was deleted (Fig. 5), indicating that *vexR* was required for *vexRAB* expression in the RND efflux mutants. This suggested the possibility that metabolites accumulated within the cell and induced *vexRAB* expression in the absence of RND-mediated efflux. This idea is consistent with recent reports in *E*. *coli* which have linked efflux to cell metabolism [40–42,47]. The finding that *vexRAB* expression was upregulated in three of the 36 tested metabolic mutants provided evidence to support this hypothesis (S1 Table). Altogether, this suggests the VexAB RND efflux system plays a role in removing excess metabolites from the cell. While *vexRAB* was induced in three of the metabolic mutants, it is also possible that other efflux systems may function in a similar or redundant role to remove other metabolites from the cell. Precedence for this was found in *E*. *coli* where the *acrEF*, *yfiK* and *aaeAB* efflux systems contributed to indole, cysteine-cystine, and p-hydroxybenzoate export, respectively [48–50]. The fact that *vexRAB* expression in the Δ*vexB* was lower than what was observed in RND negative strain JB485 supports the conclusion that other *V*. *cholerae* RND efflux systems also participate in the removal of excess metabolites from within the cell (Fig. 5).

The mutations that activated *vexRAB* expression disrupted three different metabolic pathways. This suggested that *vexRAB* expression is modulated in response to multiple different metabolites (S1 Table and Fig. 6B). The mutation of *trpB* resulted in the greatest increase in *vexRAB* expression. Since TrpB catalyzes the conversion of indole to tryptophan (Fig. 6A), this suggested that indole likely contributed to *vexRAB* induction in the *trpB* mutant (Fig. 6). Activation of *vexRAB* expression by exogenous indole supported this conclusion (Fig. 6C). In addition, both JB485 and the Δ*vexB* mutant exhibited increased susceptibility to indole. This suggested that indole was a substrate for VexAB and likely additional RND efflux systems (Table 3). Based on the fact that indole both contributed to induction, and likely functioned as a substrate of *vexRAB*, we propose that the VexAB efflux system functions to remove excess indole from within the cell before it reaches concentrations that are detrimental to cell growth. Given the broad substrate specificity of the VexAB system, combined with the observation that *vexRAB* was induced in two other metabolic mutants; we suggest that the function of VexAB in metabolic relief may extend beyond indole.

In conclusion, we have shown that *vexR* contributed to the positive regulation of *vexRAB*. VexR appears to be necessary for activation of the *vexRAB* operon in response to efflux substrates of the VexAB RND efflux system. The finding that *vexR* was necessary for positive regulation was unusual. To the best of our knowledge only ten TetR family regulators from the more than 20,000 distinct TetR family proteins have been characterized as activators [29,51–61]. However, it is possible that TetR activators may be more common than currently thought as only a fraction of the annotated TetR proteins have been characterized. We have also established a novel role for VexAB in responding to the metabolic state and efflux status of the cell. We suggest that that *vexRAB* expression is modulated in response to metabolic products and that VexAB relieves stress by preventing the accumulation of metabolic byproducts to toxic levels. Our results were similar to what has been observed in *E*. *coli* where metabolic mutants and metabolic products were found to activate the *acrAB* RND system [40–42,47]. Therefore, the ability of the RND efflux systems to alleviate stress due to excess metabolites appears to be an evolutionarily conserved mechanism.

## Supporting Information

S1 Fig Effect of VexR on the expression of *Vibrio cholerae* RND efflux systems. Overnight cultures of *Vibrio cholerae* strain JB58 (N16961 Δ*lacZ* Sm^R^) or the isogenic Δ*vexR* mutant bearing the transcriptional fusion reporters for the indicated RND efflux system were diluted 1:100 into LB broth and grown with shaking for 3.5 h at 37°C when deoxycholate (DOC) was added to 0.02%. The culture was incubated with shaking at 37°C for an additional 30 min when aliquots were collected in triplicate and β-galactosidase activity was quantified as described in the methods. (A.) *vexCD*; (B.) *vexEF*; (C.) *vexGH*; (D.) *vexIJK*; (E.) *vexLM*. The reported results are in Miller Units (MU) and are the mean ± SD of three independent experiments.
(PDF)

S2 Fig Deletion of *vexR* results in upregulation of the *Vibrio cholerae* Cpx system. The indicated strains containing a chromosomal *cpxP-lacZ* transcriptional reporter were cultured overnight in LB broth before being diluted 1:100 into fresh LB broth. The cultures were incubated an additional hour and diluted 1,000-fold into PBS. Aliquots of the diluted cultures were then inoculated onto the surface of LB agar plates containing X-gal (160 μg/mL) plus and minus 500 μM CuCl_2_ (to induce expression of the Cpx system). The plates were then incubated overnight at 37°C before being photographed.
(PDF)

S3 Fig CpxR does not affect *vexRAB* expression in a *vexR* mutant. The indicated *V*. *cholerae* N16961 strains containing a *vexRAB-lacZ* transcriptional reporter were cultured to middle logarithmic phase in LB broth when *vexRAB-lacZ* expression was quantified by a β-galactosidase assay as described in the methods. The results are the average ±SD of five independent experiments.
(PDF)

S4 Fig Episomal *vexR* expression does not provide a growth advantage to a *vexR* deletion mutant. *V*. *cholerae* N16961 Δ*vexR* containing pJB703 (pBAD18::*vexR*) or pBAD18 were grown in triplicate wells of microtiter plates containing (A) LB broth or (B) LB broth plus 0.015% deoxycholate. Arabinose (0.1%) was added to the media as indicated to induce expression of *vexR* from the arabinose regulated promoter in pBAD18. Cell growth was then monitored as the change in the optical density at 600 nm and plotted versus time as the mean ±SEM.
(PDF)

S1 Table Expression of *vexRAB* in C6706 metabolic mutants. (PDF)

S2 Table Minimum inhibitory concentration of erythromycin for *Vibrio cholerae* mutants. (PDF)
