# Supplementary material for: Substrate-Dependent Activation of the Vibrio cholerae vexAB RND Efflux System Requires vexR
Source: PLoS One. 2015 Feb 19;10(2):e0117890. doi: 10.1371/journal.pone.0117890 (PMC4335029; doi:10.1371/journal.pone.0117890)
Supplement: S1 Fig — Overnight cultures of Vibrio cholerae strain JB58 (N16961 ΔlacZ SmR) or the isogenic ΔvexR mutant bearing the transcriptional fusion reporters for the indicated RND efflux system were diluted 1:100 into LB broth and grown with shaking for 3.5 h at 37°C when deoxycholate (DOC) was added to 0.02%. The culture was incubated with shaking at 37°C for an additional 30 min when aliquots were collected in triplicate and β-galactosidase activity was quantified as described in the methods. (A.) vexCD; (B.) vexEF; (C.) vexGH; (D.) vexIJK; (E.) vexLM. The reported results are in Miller Units (MU) and are the mean ± SD of three independent experiments. (PDF) [file pone.0117890.s001.pdf]

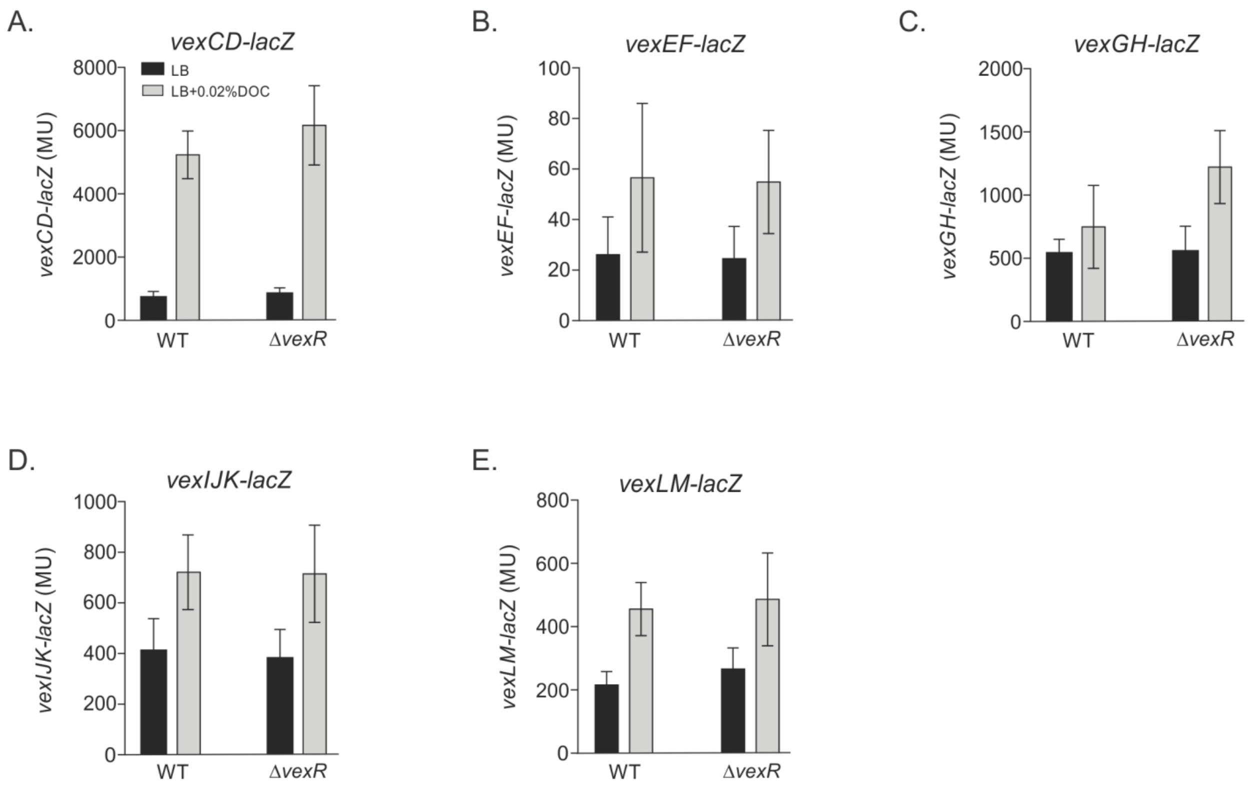

Figure S1. Effect of VexR on the expression of *Vibrio cholerae* RND efflux systems. Overnight cultures of *Vibrio cholerae* strain JB58 (N16961 Δ*lacZ* Sm^R) or the isogenic Δ*vexR* mutant bearing the transcriptional fusion reporters for the indicated RND efflux system were diluted 1:100 into LB broth and grown with shaking for 3.5 h at 37°C when deoxycholate (DOC) was added to 0.02%. The culture was incubated with shaking at 37°C for an additional 30 min when aliquots were collected in triplicate and β-galactosidase activity was quantified as described in the methods. (A.) *vexCD*; (B.) *vexEF*; (C.) *vexGH*; (D.) *vexIJK*; (E.) *vexLM*. The reported results are in Miller Units (MU) and are the mean ± SD of three independent experiments.
