# Supplementary material for: Substrate-Dependent Activation of the Vibrio cholerae vexAB RND Efflux System Requires vexR
Source: PLoS One. 2015 Feb 19;10(2):e0117890. doi: 10.1371/journal.pone.0117890 (PMC4335029; doi:10.1371/journal.pone.0117890)
Supplement: S2 Fig — The indicated strains containing a chromosomal cpxP-lacZ transcriptional reporter were cultured overnight in LB broth before being diluted 1:100 into fresh LB broth. The cultures were incubated an additional hour and diluted 1,000-fold into PBS. Aliquots of the diluted cultures were then inoculated onto the surface of LB agar plates containing X-gal (160 μg/mL) plus and minus 500 μM CuCl2 (to induce expression of the Cpx system). The plates were then incubated overnight at 37°C before being photographed. (PDF) [file pone.0117890.s002.pdf]

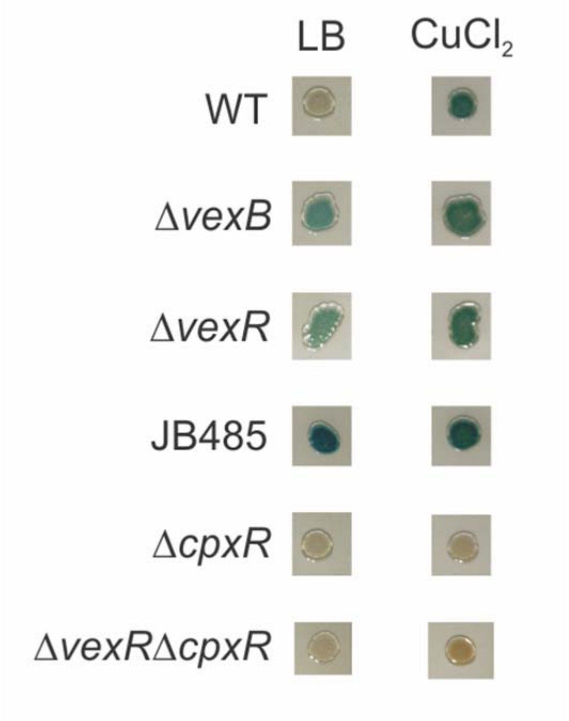

Figure S2. Deletion of *vexR* results in upregulation of the *Vibrio cholerae* Cpx system. The indicated strains containing a chromosomal *cpxP-lacZ* transcriptional reporter were cultured overnight in LB broth before being diluted 1:100 into fresh LB broth. The cultures were incubated an additional hour and diluted 1,000-fold into PBS. Aliquots of the diluted cultures were then inoculated onto the surface of LB agar plates containing X-gal (160 μg/mL) plus and minus 500 μM $CuCl_2$ (to induce expression of the Cpx system). The plates were then incubated overnight at 37°C before being photographed.
