# Supplementary material for: Substrate-Dependent Activation of the Vibrio cholerae vexAB RND Efflux System Requires vexR
Source: PLoS One. 2015 Feb 19;10(2):e0117890. doi: 10.1371/journal.pone.0117890 (PMC4335029; doi:10.1371/journal.pone.0117890)
Supplement: S3 Fig — The indicated V. cholerae N16961 strains containing a vexRAB-lacZ transcriptional reporter were cultured to middle logarithmic phase in LB broth when vexRAB-lacZ expression was quantified by a β-galactosidase assay as described in the methods. The results are the average ±SD of five independent experiments. (PDF) [file pone.0117890.s003.pdf]

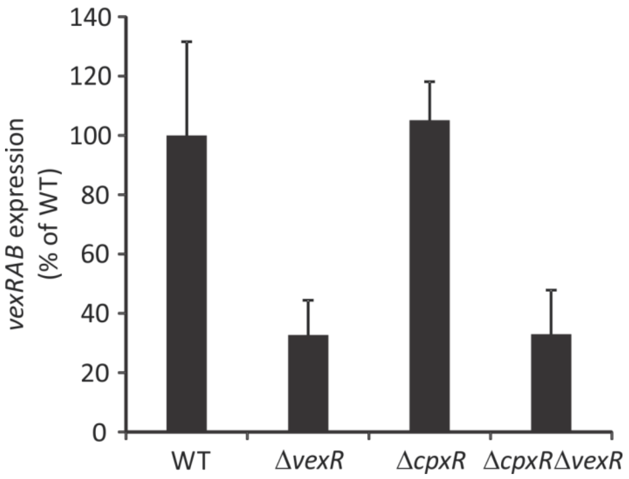

Figure S3. CpxR does not affect *vexRAB* expression in a *vexR* mutant. The indicated *V. cholerae* N16961 strains containing a *vexRAB-lacZ* transcriptional reporter were cultured to middle logarithmic phase in LB broth when *vexRAB-lacZ* expression was quantified by a β-galactosidase assay as described in the methods. The results are the average ±SD of five independent experiments.
