# Supplementary material for: Substrate-Dependent Activation of the Vibrio cholerae vexAB RND Efflux System Requires vexR
Source: PLoS One. 2015 Feb 19;10(2):e0117890. doi: 10.1371/journal.pone.0117890 (PMC4335029; doi:10.1371/journal.pone.0117890)
Supplement: S4 Fig — V. cholerae N16961 ΔvexR containing pJB703 (pBAD18::vexR) or pBAD18 were grown in triplicate wells of microtiter plates containing (A) LB broth or (B) LB broth plus 0.015% deoxycholate. Arabinose (0.1%) was added to the media as indicated to induce expression of vexR from the arabinose regulated promoter in pBAD18. Cell growth was then monitored as the change in the optical density at 600 nm and plotted versus time as the mean ±SEM. (PDF) [file pone.0117890.s004.pdf]

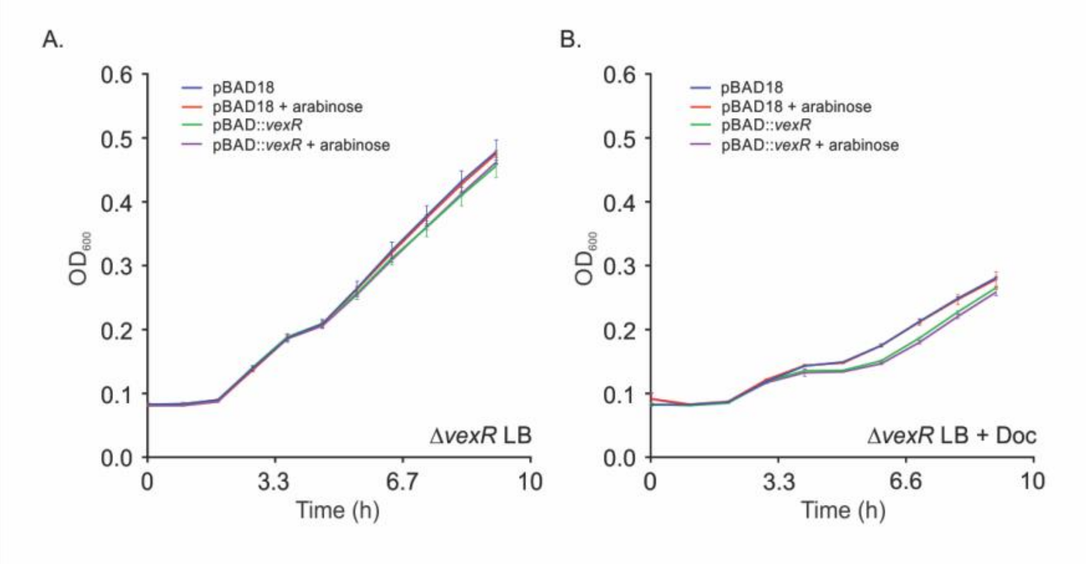

Figure S4. Episomal *vexR* expression does not provide a growth advantage to a *vexR* deletion mutant. *V. cholerae* N16961 Δ*vexR* containing pJB703 (pBAD18::*vexR*) or pBAD18 were grown in triplicate wells of microtiter plates containing (A) LB broth or (B) LB broth plus 0.015% deoxycholate. Arabinose (0.1%) was added to the media as indicated to induce expression of *vexR* from the arabinose regulated promoter in pBAD18. Cell growth was then monitored as the change in the optical density at 600 nm and plotted versus time as the mean ±SEM.
