# Supplementary material for: Substrate-Dependent Activation of the Vibrio cholerae vexAB RND Efflux System Requires vexR
Source: PLoS One. 2015 Feb 19;10(2):e0117890. doi: 10.1371/journal.pone.0117890 (PMC4335029; doi:10.1371/journal.pone.0117890)
Supplement: S1 Table — (PDF) [file pone.0117890.s005.pdf]

Table S1. Expression of *vexRAB* in C6706 metabolic mutants.

Mutated ORF (Gene Name):	Gene Product:	RLU/OD_{600} (±SD)*10^{-5}	Fold change[1]
C6706 (WT)	Wildtype parental strain	16.8(3.8)	-----
VC0027(*ilvA*)	threonine dehydratase	26.9(6.7)***	1.6
VC0051(*purK*)	phosphoribosylaminoimidazole carboxylase ATPase subunit	21.0(2.7)	1.3
VC0052(*purE*)	phosphoribosylaminoimidazole carboxylase catalytic subunit	14.4(2.3)	0.9
VC0164(*vexB*)	RND multidrug efflux pump	35.2(6.6)***	2.1
VC0374(*pgi*)	glucose-6-phosphate isomerase	18.4(0.6)	1.1
VC0384(*cysJ*)	sulfite reductase	19.5(6.4)	1.2
VC0385(*cysI*)	sulfite reductase subunit beta	10.0(0.2)***	0.6
VC0386(*cysH*)	phosphoadenosine phosphosulfate reductase	17.4(2.0)	1.0
VC0537(*cysM*)	cysteine synthase B	12.7(5.1)	0.8
VC0767(*guaB*)	inosine 5'-monophosphate dehydrogenase	13.0(2.0)	0.8
VC0774	2,3-dihydroxybenzoate-2,3-dehydrogenase	10.4(1.7)***	0.6
VC0819(*aldA*-1)	aldehyde dehydrogenase	9.6(1.3)***	0.6
VC0923	serine acetyltransferase-related protein	9.8(0.9)***	0.6
VC0968(*cysK*)	cysteine synthase A	9.4(0.5)***	0.6
VC1061	cysteine synthase	5.6(0.8)***	0.3
VC1172(*trpD*)	anthranilate phosphoribosyltransferase	37.3(0.9)***	2.2
VC1579(*almE*)	enterobactin synthetase component F-related protein	34.6(1.3)***	2.1
VC1732(*aroA*)	3-phosphoshikimate 1-carboxyvinyltransferase	10.3(0.5)***	0.6
VC1819(*aldA*-2)	aldehyde dehydrogenase	3.3(1.3)***	0.2
VC2013(*ptsG*)	PTS system glucose-specific transporter subunits IIBC	12.0(0.7)*	0.7
VC2092(*gltA*)	citrate synthase	5.9(0.3)***	0.4
VC2209(*vibF*)	nonribosomal peptide synthetase VibF	24.6(1.8)***	1.5
VC2348(*deoB*)	phosphopentomutase	7.7(3.0)***	0. 5
VC2362(*thrC*)	threonine synthase	4.1(0.5)***	0.3
VC2363(*thrB*)	homoserine kinase	6.3(3.9)***	0.4
VC2364(*thrA*)	aspartokinase I/homoserine dehydrogenase	3.6(0.2)***	0.2
VC2558(*cysC*)	adenylylsulfate kinase	11.5(9.2)***	0.7
VC2559(*cysN*)	sulfate adenylate transferase, subunit 1	2.8(0.5)***	0.2
VC2560(*cysD*)	sulfate adenylyltransferase subunit 2	15.5(2.9)	0.9
VC2649(*cysE*)	serine acetyltransferase	10.1(0.1)**	0.6
VCA0013(*malP*)	maltodextrin phosphorylase	23.9(0.6)**	1.42
VCA0014(*malQ*)	4-alpha-glucanotransferase	20.5(1.7)	1.2
VCA0765(*ybjU*)	L-allo-threonine aldolase	3.7(0.3)***	0. 2
VCA0886(*kbl*)	2-amino-3-ketobutyrate coenzyme A ligase	5.3(4.0)***	0.3
VCA0896(*zwf*)	glucose-6-phosphate 1-dehydrogenase	11.7(0.6)	0.7
VCA0987(*ppsA*)	phosphoenolpyruvate synthase	8.1(4.0)***	0.5
VCA1046(*mtlD*)	mannitol-1-phosphate 5-dehydrogenase	36.4(0.7)***	2.2

Indicated C6706 strains bearing a *vexRAB*-lux reporter were grown in 96-well plates under AKI conditions for 5h before luminescence (RLU) and OD_{600} were measured. [1]Fold change=(mutant RLU/OD_{600})/(WT RLU/OD_{600}). Two-way ANOVA with Dunnet's post-hoc test to determine statistical significance relative to WT. *=P<0.01; **=P<0.001; ***=P<0.0001.
