# Supplementary material for: Substrate-Dependent Activation of the Vibrio cholerae vexAB RND Efflux System Requires vexR
Source: PLoS One. 2015 Feb 19;10(2):e0117890. doi: 10.1371/journal.pone.0117890 (PMC4335029; doi:10.1371/journal.pone.0117890)
Supplement: S2 Table — (PDF) [file pone.0117890.s006.pdf]

Table S2. Minimum inhibitory concentration of erythromycin for *Vibrio cholerae* mutants.

**Strain:**	**MIC (ug/mL)[1.]:**	**S.D.**
WT	2.12	0.024
Δ*vexR*	0.15	0.058
Δ*vexR* Δ*cpxR*	0.11	0.031
Δ*cpxR*	2.11	0.059
Δ*vexB*	0.05	0.001
Δ*vexBDFHKM*	0.05	0.001

[1.] MICs were determined using gradient agar plates as previously described (*Infection and Immunity; 76(8):3595-3605)*.
